# Polymer Nanomedicines with pH-Triggered Pirarubicin Release: Revealing the Role of Carrier Hydrophilicity and Release Kinetics in Anticancer Performance

**DOI:** 10.1021/acs.biomac.5c01344

**Published:** 2025-09-15

**Authors:** Sára Pytlíková, Benchun Jiang, Lucie Woldřichová, Kevin Kotalík, Ladislav Androvič, Shanghui Gao, Vladimír Šubr, Anna Rumlerová, Robert Pola, Natália Podhorská, Marcela Filipová, Mingjie Zhang, Michal Pechar, Jun Fang, Richard Laga, Tomáš Etrych

**Affiliations:** † Institute of Macromolecular Chemistry, 86879 Czech Academy of Sciences, Heyrovského nám. 2, Prague 6 162 00, Czech Republic; ‡ Faculty of Pharmaceutical Sciences, 67181 Sojo University, Kumamoto 860 0082, Japan; § Department of Gastrointestinal Surgery, Shengjing Hospital of China Medical University, No. 36 Sanhao Street, Shenyang 110004, Liaoning, China; ∥ Department of General Surgery, Shengjing Hospital of China Medical University, No. 36 Sanhao Street, Shenyang 110004, Liaoning, China

## Abstract

The therapeutic efficacy of antitumor nanomedicines is influenced by numerous factors, with the most critical being the selection of an appropriate biomaterial and the use of suitable stimulus-responsive linkers. The chosen biomaterial must be biocompatible and capable of binding the drug via a linker that facilitates selective release and activation of the therapeutic effect, specifically within tumor tissue. In this study, we designed, synthesized, and compared the physicochemical and biological properties of various polymer nanomedicines, each bearing pirarubicin conjugated to water-soluble and biocompatible methacrylamide-based copolymers through pH-sensitive hydrazone bonds. Our findings indicate that the hydrophobicity and length of the linker near the hydrazone bond are crucial factors influencing the treatment efficacy of the nanomedicines. Conjugates with aminohexanoyl linkers exhibited superior drug release and enhanced antitumor activity compared with those with shorter linkers. Overall, our study highlights that the rate of drug release, governed by the linker structure, plays a pivotal role in therapeutic efficacy, while the hydrophilicity of the polymer backbone has a lesser impact.

## Graphical abstract

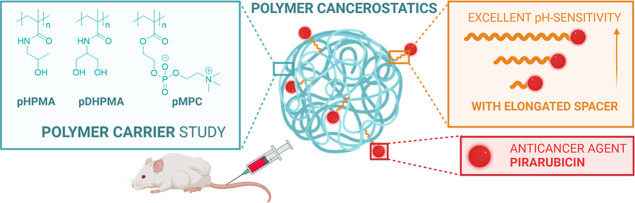

## 1 Introduction

Recently, various drug nanocarriers, including liposomes, water-soluble polymers, polymeric self-assemblies such as micelles and polymerosomes, as well as polymeric and inorganic nanoparticles, have been extensively investigated as suitable drug delivery systems (DDS) for the systemic transport of cytostatics and other bioactive molecules.^1^ One of the generally accepted approaches involves the covalent binding of a hydrophobic anticancer compound to a biocompatible and hydrophilic synthetic polymer. This conjugation approach has been generally recognized as a means to increase the solubility of the drugs, prolong the blood circulation, reduce recognition and clearance of drugs by the reticuloendothelial system, and significantly improve the pharmacokinetics and tumor accumulation.^2^ The hydrodynamic size, ranging from a few nanometers to several tens of nanometers, allows these DDS to preferentially accumulate in tumor tissue due to the so-called enhanced permeation and retention (EPR) effect.^3^ Last but not least, the functionalized polymer carrier allows for the potential attachment of a targeting moiety to enable tumor-specific delivery of the drug, as well as the incorporation of fluorescent, contrast, or radiolabels to facilitate monitoring of the system’s biodistribution.

Numerous hydrophilic polymers have been described in the literature as carriers of various antitumor drugs. Copolymers based on *N*-(2-hydroxypropyl)­methacrylamide (HPMA), polyoxazolines (Pox), and polymers with a poly­(ethylene glycol) (PEG) backbone represent the most extensively studied synthetic materials.^4^ They are primarily utilized for the delivery of various cancerostatics, particularly clinically approved anthracycline antibiotics, including doxorubicin (Dox) and pirarubicin (Pir), which are employed in the treatment of breast cancer, bladder cancer, Kaposi’s sarcoma, lymphoma, and acute lymphocytic leukemia. Recently, more hydrophilic copolymers based on *N*-(1,3-dihydroxypropyl)­methacrylamide (DHPMA) and 2-methacryloyloxyethyl phosphorylcholine (MPC) were described. These copolymers allow higher loading of the hydrophobic drugs than traditional polymer carriers based on HPMA, PEG, or Pox^5–7^ while retaining good solubility of the drug delivery system in aqueous media.

It was repeatedly demonstrated that the release of an anticancer drug from a polymer–drug conjugate in the target tissue is a prerequisite to ensure the anticancer activity of the polymer conjugate.^2^ The key point is the selection of a suitable stimuli-responsive linker between the drug and the polymer carrier, which should enable activation of the transported drug only at the given target site, i.e., the tumor tissue. These linkers can respond to changes in the physicochemical properties of the environment, e.g., changes in pH or reduction potential, or changes in the enzymatic activity within the target tissue. The rate of drug release is influenced by many factors, with two of the most important being the structure of the linker between the drug and the polymer backbone and the type of bond between the drug and the linker. The most commonly described linkers, which remain stable in the bloodstream during drug transport but undergo cleavage within the tumor environment, contain hydrolytically degradable hydrazone, ester,^8^ acetal,^9^ or imine bonds;^10^ reductively cleavable disulfide or β-thiopropionate bonds;^11^ and enzymatically cleavable peptide,^12^ carbonate,^13^ or azo bonds.^14^ However, it is important to emphasize that the selection of linkers must be guided by the desired drug release profile with respect to the characteristics of the targeted tumor tissue. A higher drug release rate does not necessarily correlate with higher therapeutic activity of the polymer–drug conjugate in vivo. This principle was demonstrated in a study evaluating the antitumor efficacy of HPMA-based polymer conjugates bearing cytarabine in vivo.^15^ The polymer-cytarabine conjugates with a slowly hydrolytically cleavable aminohexanoyl linker between the drug and the polymer backbone exhibited significantly better antitumor activity in vivo compared with analogous polymer-cytarabine conjugates with the tetrapeptide linker GFLG, which is susceptible to both chemical and enzymatic hydrolysis and releases cytarabine much faster.

In this study, we investigated several polymer conjugates with Pir attached to the polymer carrier of different structures, e.g., HPMA copolymer, DHPMA copolymer, or MPC copolymer, via pH-sensitive hydrazone bonds. To determine the optimal linker composition influencing the hydrolysis rate of the hydrazone bond, thereby facilitating Pir release and modulating tumor growth suppression in vivo, the hydrophobicity and length of the linker were systematically varied (Scheme 1). It was observed that the rate of the drug release in mild acid conditions (pH 5.0) is controlled by the detailed structure of the linker between Pir and the main polymer chain. Moreover, the drug release rate significantly affected the final antitumor activity of the polymer conjugates. In parallel to the effect of the linker, we also compared polymer carriers differing in hydrophilicity based on three different basic monomer units—HPMA, DHPMA, and 2-methacryloyloxyethyl phosphorylcholine (MPC)—both in terms of the drug release rate and antitumor activity in vivo.

**Scheme 1:**
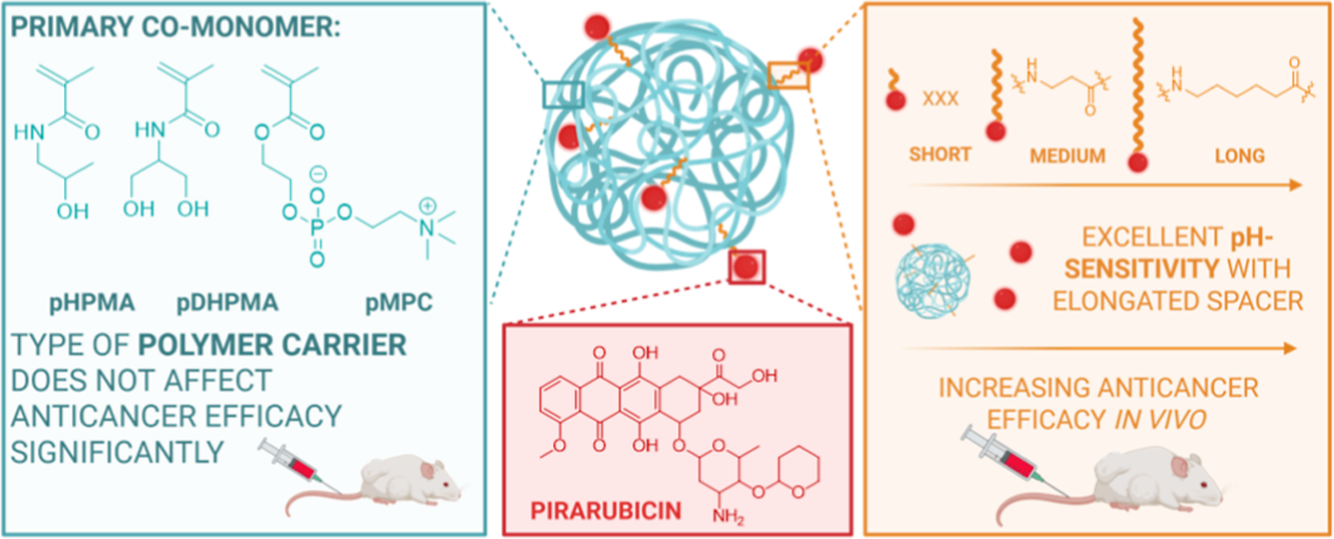
Schematic Representation Illustrating the Overall Concept of the Article

## 2 Experimental Section

### 2.1 Chemicals

Methacryloyl chloride, 2-methacryloyloxyethyl phosphorylcholine (MPC), 1-aminopropan-2-ol, 2-amino-1,3-propanediol, aminohexanoic acid, aminopropanoic acid, 2-cyano-2-propyl benzodithioate (DTB-AIBN), *tert*-butyl carbazate, 4-dimethylaminopyridine (DMAP), 1-ethyl-3-(3-(dimethylamino)­propyl)­carbodiimide (EDC), (1,1,3,3-tetramethylbutyl)­pyrocatechol, 2,5-di-*tert*-butylhydroquinone, sodium sulfate, hydrochloric acid, acetic acid, dichloromethane (DCM), tetrahydrofuran (THF), hexane, *N*,*N*-dimethylformamide (DMF), dimethyl sulfoxide (DMSO), *tert*-butyl alcohol (tBuOH), dimethylacetamide (DMA), ethyl acetate, diethyl ether, and acetone were purchased from Merck (Prague, Czech Republic). 2,2′-Azobis­(4-methoxy-2,4-dimethylvaleronitrile) (V-70) and azobis­(isobutyronitrile) (AIBN) were purchased from Wako Chemicals Europe (Neuss, Germany). Pirarubicin was obtained from Meiji Seika (Tokyo, Japan). DMSO, DMA, and tBuOH for the synthesis of semitelechelic homopolymers were dried with molecular sieves. Acetone and diethyl ether for the synthesis of semitelechelic homopolymers were dried with anhydrous sodium sulfate, distilled, and stored with molecular sieves.

### 2.2 Syntheses of Monomers and Chain Transfer Agent

#### 2.2.1 HPMA, DHPMA, MA-Ah-NHNHBoc, TTC-TT, and TTC-AIBN

*N*-(2-Hydroxypropyl)­methacrylamide (HPMA) was synthesized as described previously.^16^ *N*-(1,3-Dihydroxypropyl)­methacrylamide (DHPMA) was prepared as described in the literature.^17^ *N*-(*tert*-Butoxycarbonyl)-*N*′(6-methacrylamidohexanoyl)­hydrazine (MA-Ah-NHNHBoc) was prepared by a two-step procedure as described previously.^18^ The synthesis of a chain transfer agent for RAFT polymerization (CTA)­2-cyano-5-oxo-5-(2-thioxo-1,3-thiazolidin-3-yl)­pentan-2-yl ethyl trithiocarbonate (TTC-TT) was carried out according to the previously reported procedure.^19,20^ The CTA *S*-2-cyano-2-propyl-*S*′-ethyl trithiocarbonate (TTC-AIBN) was also performed as described previously.^21^

#### 2.2.2 *N*-(*tert*-Butoxycarbonyl)-*N*′-(6-methacrylamidopropanoyl)­hydrazine (MA-Ap-NHNHBoc)

MA-Ap-NHNHBoc was prepared in a two-step synthesis. In the first step, *N*-methacryloyl-6-aminopropanoic acid (MA-Ap-OH) was prepared by reaction of methacryloyl chloride with β-alanine as described previously.^22,23^ Then, MA-Ap-OH was reacted with *tert*-butyl carbazate as follows. MA-Ap-OH (3.2 g, 0.02 mol, 1 equiv) was dissolved in dichloromethane (80 mL), then *tert*-butyl carbazate (2.8 g, 0.021 mol, 1.05 equiv), a catalytic amount of DMAP, and a trace amount of the polymerization inhibitor, 2,5-di-*tert*-butylhydroquinone were added. Subsequently, EDC (5.0 g, 0.026 mol, 1.3 equiv) was added gradually over the course of 1 h. Then, the reaction mixture was stirred at room temperature for the next 2 h. The product was separated from the reaction mixture by extraction with ethyl acetate (3 × 30 mL), then dried with sodium sulfate overnight, filtrated, and partially evaporated. The product was crystallized from ethyl acetate with a small addition of hexane, yielding 2.6 g of white crystalline product corresponding to a conversion of 48%. Melting point: 131 °C; elemental analysis (calculated wt %/found wt %): C 53.12/52.47; H 7.80/7.73; N 15.49/15.24, ^1^H NMR (400 MHz, DMSO_d6_): δ 1.39 (s, 9H, –O­(CH_3_)_3_), 1.84 (s, 3H, –CH_3_), 2.29 (t, 2H, ^3^ *J*_HH_ = 7.28 Hz, –CH_2_−), 3.32 (s, 2H, –CH_2_−), 5.31 (q, 1H, ^2^ *J*_HH_ = 1.56 Hz, =CH_2_). 5.65 (s, 1H, =CH_2_), 7.89 (br s, 1H, –NH–), 8.71 (br s, 1H, -NHNHBoc), 9.56 (br s, 1H, –NHNHBoc) ppm.

#### 2.2.3 *N*-(*tert*-Butoxycarbonyl)-*N*′-(methacryloyl)­hydrazine (MA-NHNHBoc)

MA-NHNHBoc was synthesized by the reaction of methacryloyl chloride with *tert*-butyl carbazate as follows. *tert*-Butyl carbazate (12.6 g; 0.0957 mol; 1 equiv) was dissolved in DCM (225 mL) and sodium carbonate (12.17 g; 0.115 mol; 1.2 equiv) with a trace amount of the polymerization inhibitor (1,1,3,3-tetramethylbutyl)­pyrocatechol. The reaction mixture was cooled to 0 °C, and methacryloyl chloride (9.35 mL; 0.0957 mol; 1 equiv) dissolved in DCM (25 mL) was added dropwise while maintaining the temperature at 0 °C. When all the methacryloyl chloride was added, the reaction mixture was warmed to room temperature and stirred for the next 2 h. After the reaction, the sodium carbonate was filtered off. The product was separated from the reaction mixture by extraction with water (3 × 50 mL). Subsequently, the organic phase was dried with sodium sulfate for 1 h, filtrated, and partially evaporated under reduced pressure. The product was crystallized after the addition of hexane (100 mL), yielding 13.4 g of white crystalline product corresponding to a conversion of 70%. Melting point: 118 °C, elemental analysis (calculated wt %/found wt %): C 53.98/53.94; H 8.05/8.06; N 13.99/14.06, ^1^H NMR (400 MHz, DMSO_d6_): δ 9.65 (s, 1H, –NH–C­(O)–C–), 8.71 (s, 1H, –NH–C­(O)–O−), 5.70 (s, 1H, –C=CH_2_), 5.41 (s, 1H, –C=CH_2_), 1.86–1.84 (m, 3H, –CH_3_), 1.40 (s, 9H, −O–C–(CH_3_)_3_) ppm.

### 2.3 Syntheses of Polymers

Both semitelechelic homopolymers with thiazolidine-2-thione (TT) end groups (Scheme S1) and statistical copolymers based on HPMA, MPC, and DHPMA (Scheme 2) were prepared by RAFT polymerization. The physicochemical characteristics of all of the TT-semitelechelic homopolymers are summarized in Table 1. The molar ratio of the main comonomer (HPMA, MPC, and DHPMA) to the protected hydrazide comonomer Ma-Ah-NHNHBoc, Ma-Ap-NHNHBoc, or Ma-NHNHBoc monomers was 92:8 in all copolymerizations. The physicochemical characteristics of all of the copolymers are summarized in Table 2.

**Scheme 2:**
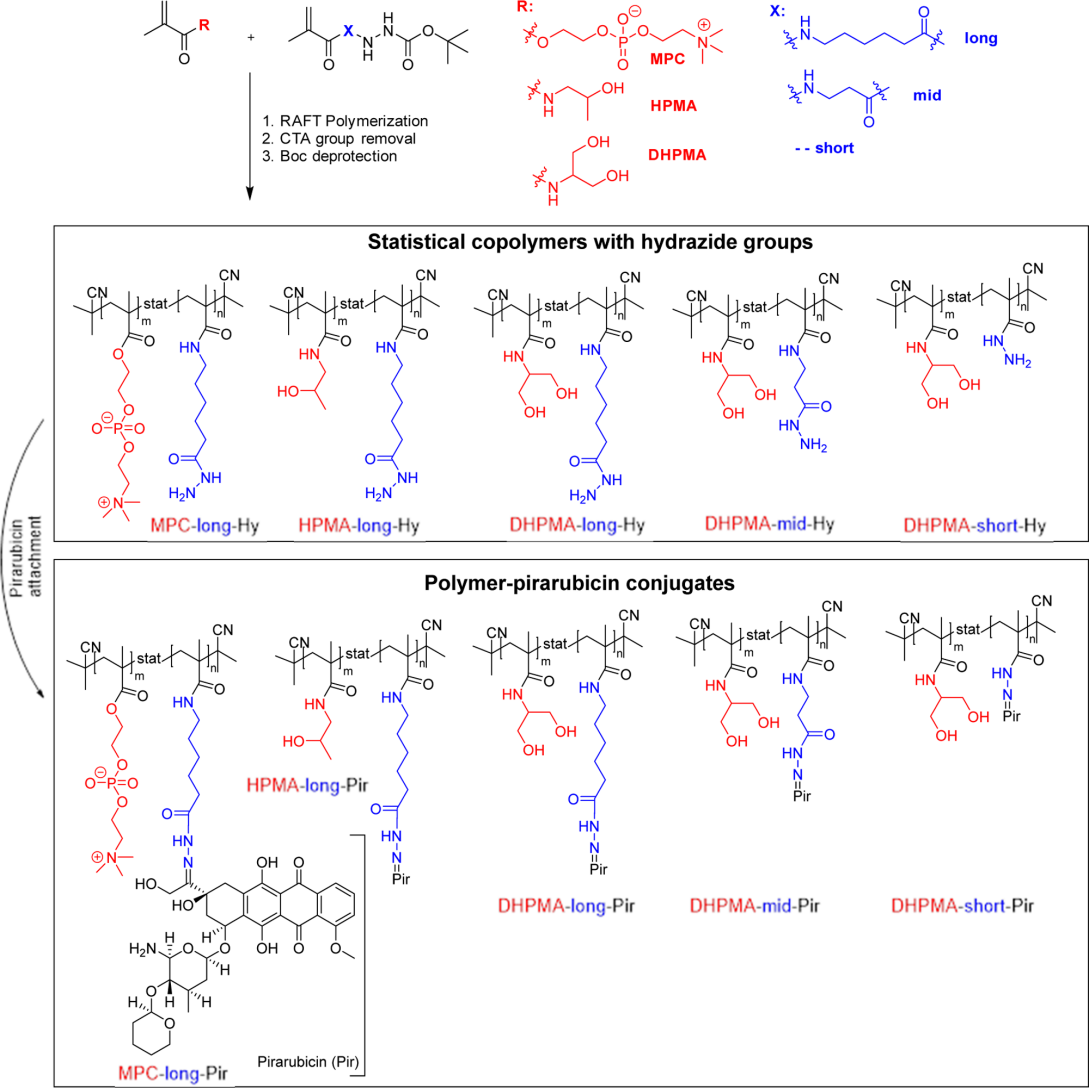
Schematic Representation of Polymer-Pir Conjugates Synthesis

**Table 1:** Physico-Chemical Characterization of Dy647-Semitelechelic Homopolymers Differing in the Polymer Carrier Structure

polymer	*M*_w precursor_ ^a^ (g mol^–1^)	*D̵*_precursor_ ^a^	wt % Dy647^b^
Dy647-poly(MPC)	27,600	1.01	3.8
Dy647-poly(HPMA)	17,800	1.13	3.0
Dy647-poly(DHPMA)	18,600	1.06	2.3

a Weight-average molecular weight (*M*_w_) and dispersity values (*D̵*) of polymer precursors were evaluated by SEC using MALS and RI detection on a Superose 6 Increase 10/300 GL column in PBS.

b Dy647 content was determined by UV–vis spectrometry at λ = 647 nm.

**Table 2:** Physico-Chemical Characterization of Polymer Precursors Differing in the Linker Length and in the Polymer Carrier Structure

polymer	*M*_w_ (g mol^–1^)^a^	*D̵* ^a^	*D*_H_ (nm)^b^	mol % hydrazides (Hy)^c^
**MPC-long-Hy**	31,200	1.10	7.3	7.0
**HPMA-long-Hy**	30,900	1.16	6.9	6.3
**DHPMA-long-Hy**	32,100	1.12	7.5	5.6
**DHPMA-mid-Hy**	27,500	1.05	7.9	7.7
**DHPMA-short-Hy**	28,100	1.08	6.5	8.3

a Weight-average molecular weight *M*_w_ and dispersity *D̵* were evaluated by SEC using MALS and RI detection on a Superose 6 Increase 10/300 GL column in PBS.

b Hydrodynamic diameters *D*_H_ of the polymers were determined by DLS in PBS at 25 °C; the polymer concentration was 1 mg mL^–1^.

c The Content of free hydrazide groups was evaluated by the TNBSA assay by UV–vis spectrometry.

#### 2.3.1 TT-Semitelechelic TT-Poly­(MPC), TT-Poly­(HPMA), and TT-Poly­(DHPMA)

For the synthesis of TT-poly­(MPC), a mixture of MPC monomer (500 mg, 3.5 mmol), chain transfer agent (DTB-AIBN) (5.9 mg, 29.7 μmol), and azo-initiator (AIBN) (2.2 mg, 13.3 μmol) was dissolved in a mixture of dry MeOH and DMSO in a ratio 8/2 (1.5 mL). The final monomer concentration was 0.8 M with a molar ratio of monomer/CTA/initiator of 260/2/1. The reaction mixture was bubbled with argon and polymerized in a sealed glass ampule at 70 °C for 16 h. The crude polymer was isolated from the reaction mixture by precipitation in a mixture of acetone and diethyl ether in a ratio of 3/1 (40 mL) and reprecipitated from methanol in a mixture of acetone and diethyl ether in a ratio of 3/1 (40 mL). After centrifugation and drying under a vacuum, 401 mg of DTB-poly­(MPC) was obtained in the form of pink powder. In the next step, the TT end groups were introduced by reacting dithiobenzoate (DTB) end groups of DTB-poly­(MPC) (150 mg, 5 μmol) with azoinitiator ACVA-TT (25 mg, 152 μmol) at 80 °C for 4 h in a mixture of dry methanol and DMSO in a ratio of 78/22 (1.5 mL). After cooling to 20 °C, the reaction mixture precipitated into acetone (40 mL) and was reprecipitated from MeOH in acetone (40 mL). The reaction mixture was purified by column chromatography using a Sephadex LH-20 column in dry MeOH. The polymer was isolated by precipitation into diethyl ether (40 mL). The solid content was centrifuged and dried under a vacuum to give 103 mg of a yellow amorphous solid.

The TT-semitelechelic homopolymers based on HPMA and DHPMA were synthesized as follows: For the synthesis of TT-poly­(HPMA), a mixture of HPMA monomer (150 mg, 1.05 mmol), chain transfer agent (TTC-TT) (3.32 mg, 9.11 μmol), and azo-initiator (V-70) (1.41 mg, 4.55 μmol) was dissolved in a mixture of dry tBuOH and DMA in a ratio of 9/1 (1.3 mL). The final monomer concentration was 0.8 M with a molar ratio of monomer/CTA/initiator of 230/2/1. The reaction mixture was bubbled with argon and polymerized in a sealed glass ampule at 30 °C for 72 h. For the synthesis of TT-poly­(DHPMA), a mixture of DHPMA monomer (150 mg, 9.43 mmol), chain transfer agent (TTC-TT) (3.21 mg, 8.81 μmol), and azo-initiator (V-70) (1.36 mg, 4.40 μmol) was dissolved in dry DMSO (1.2 mL). The final monomer concentration was 0.8 M with a molar ratio of monomer/CTA/initiator of 215/2/1. The reaction mixture was bubbled with argon and polymerized in a sealed glass ampule at 30 °C for 72 h.

All of the polymers were isolated from the reaction mixture by double precipitation in a mixture of dry acetone and dry diethyl ether 2/1 and dried under reduced pressure. In the next step, the TTC end groups were removed by reaction with an excess of AIBN (20 wt % relative to the weight of the polymer) at 80 °C for 3 h in DMSO,^24^ and the polymers were isolated by double precipitation in a mixture of dry acetone and dry diethyl ether 2/1 and dried under reduced pressure to obtain slightly yellow powder corresponding to yields at about 80%.

#### 2.3.2 Poly­(MPC-*co*-MA-Ah-NHNHBoc) (**MPC-long-Hy**)

Statistical copolymer **MPC-long-Hy** was prepared by RAFT polymerization. A mixture of monomer Ma-Ah-NHNHBoc (265.3 mg, 0.9 mmol), chain transfer agent (CTA-COOH) (15.9 mg, 28.4 μmol), and azo-initiator (AIBN) (4.7 mg, 57 μmol) was dissolved in dry DMA (1.03 mL). The MPC monomer (1000 mg, 3.4 mmol) was dissolved in dry MeOH (3.67 mL). Solutions were combined to achieve a total monomer concentration of 0.9 M and a molar ratio M/CTA/I (150/2/1). The reaction mixture was bubbled with argon and polymerized in a sealed glass ampule at 70 °C for 16 h. The polymers were isolated from the reaction mixture by double precipitation in a mixture of acetone and diethyl ether 3/1 and dried under reduced pressure. 350 mg of poly­(MPC-*co*-MA-Ah-NHNHBoc) was obtained in the form of a pink powder. In the next step, the DTB end groups were removed by reaction with an excess of AIBN (20 wt % relative to the weight of the polymer) at 80 °C for 2 h in the MeOH,^25^ and the polymer was isolated by double precipitation into acetone/diethyl ether 3/1. Subsequently, Boc-deprotection of the hydrazide groups was performed by incubating the polymer in a 10% aqueous solution at 100 °C for 2 h. The product was isolated from the reaction mixture by lyophilization, yielding 150 mg.

#### 2.3.3 Poly­(HPMA-*co*-MA-Ah-NHNHBoc) (**HPMA-long-Hy**) and poly­(DHPMA-*co*-MA-Ah-NHNHBoc) (**DHPMA-long-Hy**)

Statistical copolymers **HPMA-long-Hy** and **DHPMA-long-Hy** were prepared by RAFT polymerization, as described previously.^7^ Briefly, HPMA (DHPMA) and MA-Ah-NHNHBoc monomers were diluted in a mixture of tBuOH and DMA in a ratio of 9/1 (DMF/water pH 1 in a ratio of 6/4), and then CTA (TTC-AIBN) and azo-initiator (V-70) were added. The final molar ratio of monomers/CTA/initiator was 490/2/1 (320/2/1). The polymerization took place at 40 °C for 24 h (30 °C for 72 h). The polymers were isolated from the reaction mixture by double precipitation in a mixture of acetone and diethyl ether 2/1 and dried under reduced pressure.

#### 2.3.4 Poly­(DHPMA-*co*-MA-Ap-NHNH_2_) (**DHPMA-mid-Hy**) and poly­(DHPMA-*co*-MA-NHNH_2_) (**DHPMA-Short-Hy**)

Synthesis of polymeric precursor **DHPMA-mid-Hy** proceeded in three steps: DHPMA (200 mg, 1.26 mmol) and MA-Ap-NHNHBoc (30 mg, 0.11 mmol, corresponding to a molar ratio of 92/8 comonomers) were dissolved in a mixture of DMF and water acidified with HCl to pH ≈ 1 (6/4) to achieve a total monomer concentration of 0.8 M. Polymerization was carried out at 30 °C for 72 h using V-70 (1.32 mg, 4.27 μmol) as the initiator and TTC-AIBN (1.75 mg, 8.54 μmol) as a chain transfer agent (CTA). The ratio of monomers/TTC-AIBN/V-70 = 320/2/1 was used. The polymer containing the trithiocarbonate (TTC) end groups was isolated from the reaction mixture by precipitation into excess of ethyl acetate and purified by reprecipitation from DMF to ethyl acetate to give 193 mg (84%) of product as a faint yellow powder. In the next step, the TTC end groups were removed by reaction with an excess of AIBN (20 wt % relative to the weight of the polymer) at 80 °C for 3 h in DMSO,^24^ and the polymer was isolated by double precipitation into ethyl acetate. The NMR spectrum of the polymer precursor is shown in Figure S1. Subsequently, Boc-deprotection of the hydrazide groups was performed by incubating the polymer in a 10% aqueous solution at 100 °C for 3 h. The product was isolated from the reaction mixture by lyophilization, yielding 160 mg.^7^ Synthesis of the polymeric precursor **DHPMA-short-Hy** was proceeded analogically to **DHPMA-mid-Hy** with use of MA-NHNHBoc instead of MA-Ap-NHNHBoc.

### 2.4 Synthesis of Dy647-Semitelechelic Homopolymers

All fluorescently labeled homopolymers were synthesized analogically, as follows (Scheme S1): 40 mg of the TT-semitelechelic homopolymer with removed TTC end-groups (corresponding to 1.5 μmol of TT groups) was dissolved in dried DMSO (400 μL). Then, one-third of Dy647-NH_2_ (1.5 mg, 2.19 μmol) and DIPEA (5 μL, 29 μmol) were added to the reaction mixture. The second and third parts were added 20 min and 1 h later, respectively. The reaction proceeded for two more hours. The products were isolated from the reaction mixture by precipitation in diethyl ether followed by drying under reduced pressure and purification on a calibrated PD-10 column with subsequent lyophilization, yielding 35 to 38 mg of Dy647-labeled homopolymers.

### 2.5 Synthesis of the Polymer-Pir Conjugates

All polymer-Pir conjugates (except **MPC-long-Pir**) were synthesized similarly by the following procedure^7,26^ (Scheme 2): 60 mg of polymer (20 μmol of hydrazide groups) and Pir (7.2 mg, 12 μmol) were dissolved in anhydrous DMSO (480 μL) with acetic acid (72 μL). The reaction proceeded in the dark for 24 h at 25 °C for **HPMA-long-Pir** and **DHPMA-long-Pir** and 72 h for **DHPMA-mid-Pir** and **DHPMA-short-Pir**. The polymer–drug conjugates were precipitated into an excess of anhydrous ethyl acetate and purified from the unreacted Pir by reprecipitation from DMF (DHPMA-based polymer conjugates) or methanol (HPMA-based polymer conjugates) into ethyl acetate. The resulting polymer-Pir conjugates were obtained in the form of a red powder, reaching yields of 95%. **MPC-long-Pir** was synthesized analogously by using MeOH as a solvent. The reaction took place in the dark for 24 h at 25 °C. The product was purified by SEC on Sephadex LH-20 in MeOH and precipitated to dry diethyl ether. The physicochemical characteristics of the polymer-Pir conjugates are summarized in Table 3. The NMR spectrum of the polymer conjugate **DHPMA-mid-Pir** is shown in Figure S2.

**Table 3:** Physico-Chemical Characterization of Polymer-Pir Conjugates Differing in the Linker Length and in the Polymer Carrier Structure

Polymer	*M*_w_ (g mol^–1^)^a^	*D̵* ^a^	*D*_H_ (nm)^b^	wt % Pir^c^
**MPC-long-Pir**	40,500	1.24	8.8	9.1
**HPMA-long-Pir**	44,700	1.28	9.0	9.8
**DHPMA-long-Pir**	48,700	1.36	9.0	11.7
**DHPMA-mid-Pir**	43,200	1.19	8.4	9.7
**DHPMA-short-Pir**	42,000	1.20	8.2	8.9

a Weight-average molecular weight *M*_w_ and dispersity *D̵* were evaluated by SEC using MALS and RI detection on a Superose 6 Increase 10/300 GL column in PBS.

b Hydrodynamic diameters *D*_H_ of the polymers was determined by DLS in PBS at 25 °C; the polymer concentration was 1 mg mL^–1^.

c Pir content was determined by UV–vis spectrometry at λ = 488 nm.

### 2.6 Physico-Chemical Characterization of the Polymer Conjugates

#### 2.6.1 Determination of Molecular Weights of Polymers

Determination of weight-average molecular weight (*M*_w_), number-average molecular weight (*M*_n_), and dispersity (*D̵*) of the copolymers was performed using the HPLC system (Shimadzu, Kyoto, Japan) on size exclusion columns, Superose 12 10/300 GL, or Superose 6 Increase 10/300 GL, using isocratic elution in 0.05 M phosphate buffer with 0.15 M NaCl, pH 7.4 (PBS) equipped with external multiangle light scattering (MALS) detector DAWN Helios-II, viscosimetric detector ViscoStar III, and refractometric (RI) detector Optilab (all from Wyatt Technology Corp., Goleta, CA, USA) at a flow rate of 0.5 mL/min. The data were analyzed using the ASTRA VI software, and the refractive index increment values (d*n*/d*c*) of 0.163 mL g^–1^ for DHPMA copolymers, 0.167 mL g^–1^ for HPMA copolymers, and 0.125 mL g^–1^ for MPC copolymers were applied for the calculation of *M*_n_, *M*_w_, and *D̵*.

#### 2.6.2 Determination of Hydrodynamic Diameters (*D*_H_)

Hydrodynamic diameters (*D*_H_) of the polymers were measured by dynamic light scattering (DLS) and static light scattering using a Nano-ZS instrument Zetasizer (ZEN3600, Malvern, Malvern, UK) in a phosphate buffer (0.1 M, with 0.05 M NaCl; polymer concentrations: 1 mg mL^–1^) at 25 °C. The intensity of scattered light was detected at angle θ = 173°. The wavelength of the laser was 632.8 nm. The values were the means of at least five independent measurements.

#### 2.6.3 Determination of the Dy647 Content

The content of the fluorescent dye Dy647 in the polymer conjugates was determined by UV–vis spectroscopy in DMSO using the molar absorption coefficient ε_Dy647; DMSO_ = 250,000 L mol^–1^ cm^–1^ (λ_max_ = 647 nm).

#### 2.6.4 Determination of the Hydrazide Group Content

The content of the hydrazide groups was determined by a colorimetric analysis using the TNBSA assay, as described previously.^27^ Briefly, the water solution of TNBSA was added to a polymer solution in 0.01 M borate buffer (pH = 8.3). The resulting concentration of the polymer was 0.2 mg mL^–1^ in 0.01 M borate buffer. The absorbance was measured on a UV–vis spectrometer (Jenway, London, UK) at 500 nm a using molar absorption coefficient of ε_hydrazide_ = 17,200 L mol^–1^ cm^–1^.

#### 2.6.5 Determination of the Pir Content

The content of the Pir in polymer conjugates was determined by UV–vis spectroscopy in DMSO using the molar absorption coefficient ε_Pir; DMSO_ = 11,400 L mol^–1^ cm^–1^ (λ_max_ = 488 nm). The absence of unbound Pir in the purified polymer conjugates was verified by SEC using a Shimadzu HPLC system equipped with a TSKgel G3000SW_XL_ (300 mm × 7.8 mm; 5 μm) column and a UV–vis detector Shimadzu SPD-10AVvp (488 nm). The eluent was a mixture of methanol (50 vol %) and PBS, and the flow rate was 0.5 mL min^–1^.

#### 2.6.6 Determination of the Rate of Pir Release from Polymer Conjugates

Polymer-Pir conjugates were incubated in phosphate buffers (0.15 M) at pH 5.0 or 7.4 at 37 °C using a concentration of 2 mg mL^–1^. The amount of released drug was determined by SEC (at predetermined intervals of 0, 40, 120, 200, 320, 480, and 720 min and 24 h) using the Shimadzu HPLC system equipped with a TSKgel G3000SW_XL_ column (300 × 7.8 mm; 5 μm) and a UV–vis detector Shimadzu SPD-10AVvp (488 nm). The eluent was methanol (50 vol %) with PBS (pH 7.4), and the flow rate was 0.5 mL min^–1^. The content of the released Pir was calculated from the area of the peaks corresponding to polymer-bound and free Pir, respectively, at 488 nm.

### 2.7 Biological Evaluation

#### 2.7.1 Confocal Microscopy

Human pancreatic cancer cells PANC-1 were seeded in 8-well plates, and after 24 h, cells were incubated with the respective polymers (1 μg/mL) for 1, 6, and 24 h. After this time, cells were incubated with Hoechst 33342 nuclear stain (2 μg/mL; ThermoFisher Scientific) and CellMaskTM plasma green (1× working solution according to the manufacturer; Invitrogen) for 15 min. After incubation, cells were washed with PBS and fixed in 150 μL of 4% paraformaldehyde and incubated at 20 °C for 15 min. After fixation, the cells were washed again in PBS. Images were acquired by using an Olympus FV10-SPD confocal microscope (Olympus Life Science).

#### 2.7.2 In Vitro Cytotoxicity

In vitro cytotoxicity assay was carried out using the MTT method with mouse colon carcinoma C26 cells. Cells were cultured in PRMI-1600 with 10% fetal bovine serum (FBS, Nichirei Biosciences INC., Tokyo, Japan) at 37 °C under 5% CO_2_. The cells (5000 cells/well) were seeded in a 96-well plate and cultured for 24 h, and the test samples were then applied to the cells at indicated concentrations. The cells were further cultured for 48 h, after which the MTT assay was performed, and the cytotoxicity was quantified by the percentage of surviving cells compared to the untreated control.

#### 2.7.3 In Vivo Biodistribution Study

Male ddY mice (6 weeks old) were obtained from SLC (Shizuoka, Japan) and housed under controlled conditions (22 ± 1 °C, 55 ± 5% relative humidity) with a 12 h light/dark cycle. All experiments were approved by the animal ethics committees and conducted in accordance with the Laboratory Protocol for Animal Handling of Sojo University.

To establish the S180 solid tumor model, we subcutaneously injected mouse sarcoma S180 cells (2 × 10^6^), maintained as ascites in the abdominal cavity of mice, into the dorsal skin of ddY mice. Tumors were allowed to grow for 8–10 days until they reached approximately 10 mm in diameter, at which point polymer-Pir conjugates were intravenously (i.v.) administered via the tail vein at a dose of 2.5 mg/kg (Pir equivalent). 24 h after injection, the mice were euthanized, and blood was collected from the inferior vena cava. Serum was obtained by centrifugation (2000*g*, 15 min). Tumor tissues, as well as normal tissues (liver and kidney), were also collected. Each tissue sample was homogenized in PBS (900 μL/100 mg tissue), followed by centrifugation (8000*g*, 15 min) to obtain tissue homogenates. To 0.5 mL of homogenate, 50 μL of 10 M HCl (final concentration: 1 M) was added, followed by 30 s of sonication. The mixture was then incubated at 50 °C for 1 h to hydrolyze Pir derivatives. The aglycone was extracted with 1 mL of chloroform, vigorously vortexed (∼1 min), and centrifuged (12,000 rpm, 5 min). 0.5 mL of the chloroform phase was transferred to a new tube and evaporated to dryness. The residue was dissolved in the HPLC mobile phase and analyzed using a Hitachi HPLC system (Chromaster 5110, Hitachi High-Tech Science Corporation, Tokyo, Japan) equipped with a 5410 UV detector (488 nm). The column used was a COSMOSIL 5C8-MS (4.6 mm × 150 mm) (Nacalai Tesque, Kyoto, Japan), maintained at 40 °C. The mobile phase consisted of 33% acetonitrile and 67% 0.1 M sodium acetate buffer (pH 5.0), with a flow rate of 1.2 mL min^–1^.

#### 2.7.4 Antitumor Efficacy In Vivo

The mouse sarcoma S180 solid tumor model, as described above, was used for the in vivo antitumor assay. When tumors reached approximately 6–8 mm in diameter, polymer-Pir conjugates were administered intravenously (i.v.) via the tail vein at a dose of 2.5 mg/kg (Pir equivalent). Control mice received an equal volume of saline. Tumor volume and body weight were measured every 2–4 days. Tumor volume (mm^3^) was calculated using the formula (*W* ^2^ × *L*)/2, where *W* is the tumor width and *L* is the tumor length. Mice were euthanized by isoflurane inhalation at saturated vapor pressure when tumors reached 2000 mm^3^.

#### 2.7.5 Statistical Evaluation

All results are expressed as the mean ± SEM. Differences between two groups were evaluated using Student’s *t*-test, while analysis of variance (ANOVA) was used for significance testing. A *P* value of less than 0.05 was considered statistically significant.

## 3 Results and Discussion

### 3.1 Synthesis of Dy647-Semitelechelic Homopolymers

In the first step, we synthesized analogous TT-semitelechelic homopolymers with similar molecular weights (Table 1, Scheme S1). The molar ratio of the monomer to CTA and initiator was adjusted accordingly. In the second step, a Dy647 dye with a free amino group was conjugated to the TT groups to obtain fluorescently labeled homopolymers. The fluorescent dye was added to the reaction mixture in three portions to achieve a maximal conversion.

### 3.2 Synthesis of Polymer-Pir Conjugates

All the polymer precursors containing Boc-protected hydrazide groups were synthesized via controlled radical copolymerization (RAFT technique) of hydrophilic monomers, e.g., HPMA, DHPMA, and MPC, with Boc-protected hydrazide comonomers (Scheme 2). The RAFT technique was employed with the aim to synthesize precisely defined materials with low dispersity and properly set molecular weight. All polymer precursors had a molecular weight of about 30,000 g mol^–1^ and a dispersity of 1.10 (see Table 2), which are suitable characteristics for polymer systems delivering the drug to the tumor tissue and subsequent elimination from the organism after they have fulfilled their role as a carrier. Next, the Boc groups were removed by heating the aqueous polymer solutions, and the resulting polymer hydrazides were reacted with Pir in methanol or DMSO to yield the target Pir-polymer conjugates containing hydrazone bonds between the keto groups on a drug and the hydrazide groups on a polymer carrier (Scheme 2). SEC chromatograms of all polymer precursors and polymer-Pir conjugates showing a narrow distribution are depicted in Figure S3A,B.

Interestingly, the rate of hydrazone bond formation was influenced by the structure of the polymer hydrazides. The highest reaction rate and conversion were observed for hydrazides with a **long** linker derived from aminohexanoic acid, followed by hydrazides with medium-length (**mid**) aminopropanoic acid linkers. In contrast, hydrazides directly attached to methacryloyl units (**short** linker) exhibited the lowest reactivity in terms of hydrazone formation. The type of the main monomer unit of the copolymers had no significant effect on the rate of Pir binding to the linker; the reaction proceeded almost quantitatively within 24 h. All polymer-Pir conjugates had similar molecular weights *M*_w_ (40–49 kg mol^–1^) and hydrodynamic diameters *D*_H_ (8.2–9.0 nm), indicating that they are in the aqueous solution in the form of individual soluble polymer coils that do not associate in any supramolecular assemblies.

### 3.3 Hydrolytic Release of Pir from Polymer Conjugates

The release of the drug molecule from the polymer carrier is a prerequisite for the biological activity of the polymer–drug conjugates in vivo. We hypothesized that the rate of Pir release could depend on the structure of the linker between the hydrazone bond and the polymer backbone and on the type of major comonomer unit of the copolymer, e.g., HPMA, DHPMA, or MPC Additionally, we focused on investigating how the antitumor efficacy of the polymer–drug conjugates correlates with the drug release rate.

Polymer-Pir conjugates with varying linker lengths between the drug and the DHPMA-based polymer backbone (Table 3) were incubated in aqueous buffers at pH 5.0 and 7.4, mimicking the endosomal and bloodstream environments, respectively. The drug release rate was evaluated using SEC in the organic phase by analyzing the areas of free and polymer-bound Pir (Figure 1).

**Figure 1:**
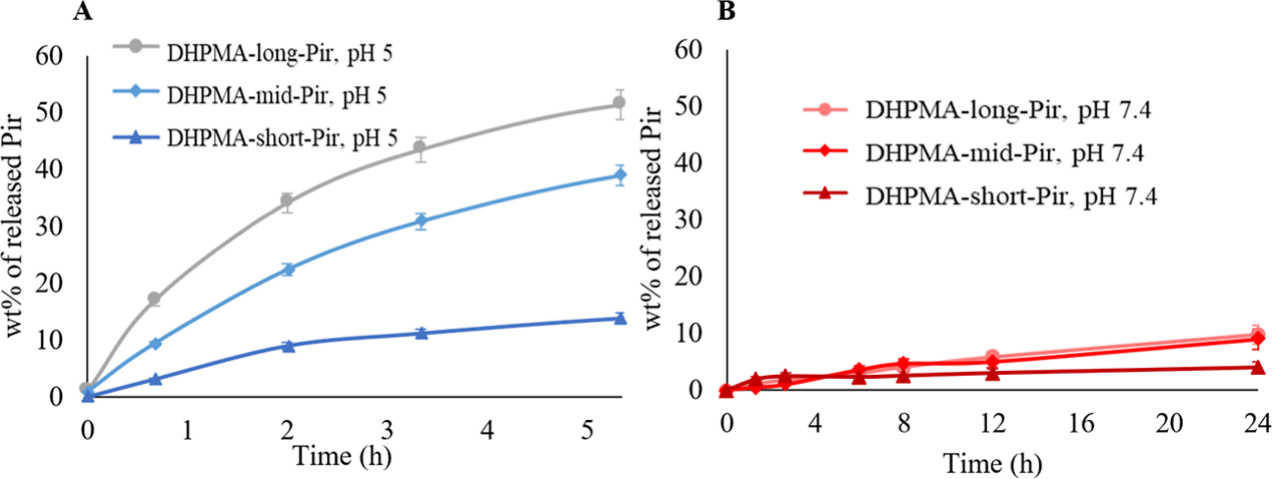
Rate of the Pir release from polymer-Pir conjugates differing in the linker length in pH 5.0 (A) and pH 7.4 (B). **DHPMA-long-Pir**, pH 5.0 (gray circle), **DHPMA-mid-Pir**, pH 5.0 (light blue square), **DHPMA-short-Pir**, pH 5.0 (blue triangle), **DHPMA-long-Pir**, pH 7.4 (pink circle), **DHPMA-mid-Pir**, pH 7.4 (red square), and **DHPMA-short-Pir**, pH 7.4 (brown triangle). *n* = 3.

The highest rate of Pir release at pH 5.0 was observed for conjugate **DHPMA-long-Pir**, containing an aminohexanoyl linker, with 50% of the drug released after 5 h. Conjugate **DHPMA-mid-Pir**, with an aminopropanoyl linker, released approximately 40% of the drug within the same period. Conjugate **DHPMA-short-Pir**, which lacked a linker between methacroyl residue and hydrazone bond, exhibited the lowest drug release rate, with only 12% of the drug released after 5 h. At pH 7.4, the drug release rates were significantly lower: 9% for conjugates **DHPMA-long-Pir** and **DHPMA-mid-Pir**, and 4% for conjugate **DHPMA-short-Pir**. Shortening the linker between the DHPMA polymer backbone and hydrazone bond thus strongly reduced the susceptibility of the conjugate to hydrolysis. However, the exact explanation of this behavior remains unclear.

These results align with literature,^26^ confirming the relative stability of polymer–drug conjugates during circulation in the bloodstream. The stability of nanomedicines in a pH 7.4 mimicking bloodstream conditions allows delivery of the cargo to the tumor tissue. Indeed, subsequent fast drug release in acidic environments (pH 5.0) causes activation of Pir in tumor tissues or cells.

To further investigate the influence of polymer carrier structure on drug release, we studied three polymers with varying hydrophilicity, **MPC-long-Pir**, **HPMA-long-Pir**, **and DHPMA-long-Pir** (Table 3), and an aminohexanoyl linker between the methacroyl residue and hydrazone bond. The results (Figure 2) revealed that the initial hydrolysis rates were similar across all conjugates. After 6 h, the highest Pir release (65%) was observed for the conjugate **HPMA-long-Pir**, followed by the conjugate **MPC-long-Pir** (50%) and the conjugate **DHPMA-long-Pir** (50%). Importantly, both **HPMA-long-Pir** and **DHPMA-long-Pir** showed excellent pH-responsiveness with only 6–8% Pir released at 7.4 after 24 h. On the contrary, **MPC-long-Pir** showed a much higher release rate at neutral conditions, releasing 25% of Pir in 24 h. We hypothesize that this lower stability of the hydrazone bond in **MPC-long-Pir** could be ascribed to the higher hydrophilicity of the carrier, enabling enhanced access of water molecules to the hydrazone bond.

**Figure 2:**
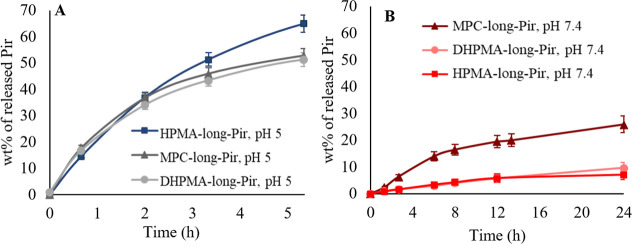
Rate of the Pir release from polymer-Pir conjugates differing in the carrier structure at pH 5.0 (A) and pH 7.4 (B). **HPMA-long-Pir**, pH 5.0 (blue square), **MPC-long-Pir**, pH 5.0 (dark gray triangle), D**HPMA-long-Pir**, pH 5.0 (gray circle), **MPC-long-Pir**, pH 7.4 (brown triangle), D**HPMA-long-Pir**, pH 7.4 (pink circle), and **HPMA-long-Pir**, pH 7.4 (red square). *n* = 3.

Based on the release rate analysis, the HPMA-based polymer-Pir conjugates demonstrated the most pronounced pH-sensitive behavior, making them ideal candidates for an advanced stimuli-sensitive drug delivery system.

### 3.4 Internalization of Homopolymers in Cancer Cell Lines

To quantify the amount of homopolymers internalized into the cells through the cell membrane, fluorescently labeled polymers containing Dy647 fluorescent dye (Table 1) were utilized. All homopolymers exhibited similar molecular weight and dispersity, ensuring that membrane translocation was influenced solely by the hydrophilic environment of the polymer coils. Additionally, due to their semitelechelic character, these polymers are well-suited for studying internalization while minimizing the impact of the dye on the process, as each polymer chain carries at most one dye molecule.

We demonstrated that all homopolymers were internalized in comparable amounts (Figures 3 and S4) over the measured time intervals (6 and 24 h). This outcome was expected, as all polymers are highly hydrophilic and well soluble in water, exhibiting similar solution behavior. This is further supported by confocal microscopy images and the corresponding quantitative analysis, as shown in Figure S5.

**Figure 3:**
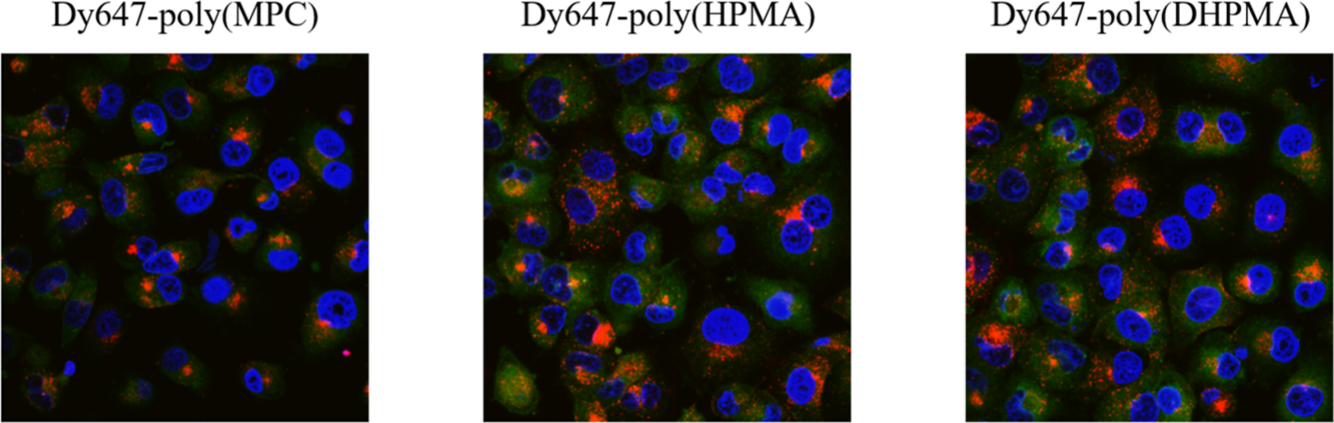
Confocal microscopy images of PANC-1 cells after 24 h of incubation with fluorescently labeled homopolymers (red) at a concentration of 1 μg/mL. Cell nuclei were stained with Hoechst 33342 nuclear stain (2 μg/mL, blue) and cell membranes with CellMaskTM (green).

The enhanced cellular uptake of MPC-based polymers is likely due to the phosphatidylcholine motif present in their structure, which mimics biological membrane lipids and thereby facilitates interactions with membrane components, including choline transporters and acetylcholine receptors. These interactions may stimulate receptor-mediated endocytosis, consequently accelerating cellular uptake.^28,29^ In contrast, HPMA and DHPMA polymers lack specific motifs capable of interacting with membrane receptors or transporters, and their uptake occurs predominantly through passive, nonspecific endocytosis or pinocytosis, processes that proceed more slowly compared with the receptor-assisted pathways observed for MPC polymers.

### 3.5 In Vitro Cytotoxicity of Polymer-Pir Conjugates in Cancer Cell Lines

To confirm and compare the in vitro cytotoxicity of different polymer-Pir conjugates, an MTT assay was performed using mouse colon cancer C26 cells. As shown in Figure 4, dose-dependent cytotoxicity was confirmed for all samples. When we compared the different polymer conjugates, we found that **DHPMA-short-Pir** showed a lower cytotoxicity than other polymer conjugates, whereas other polymer conjugates exhibited similar cytotoxicity profiles (Figure 4). These findings are consistent with the release profiles of the polymer-Pir conjugates as described in Figures 1 and 2, suggesting that the release dynamics significantly affect the cytotoxicity and therapeutic effect of the polymer-Pir conjugates.

**Figure 4:**
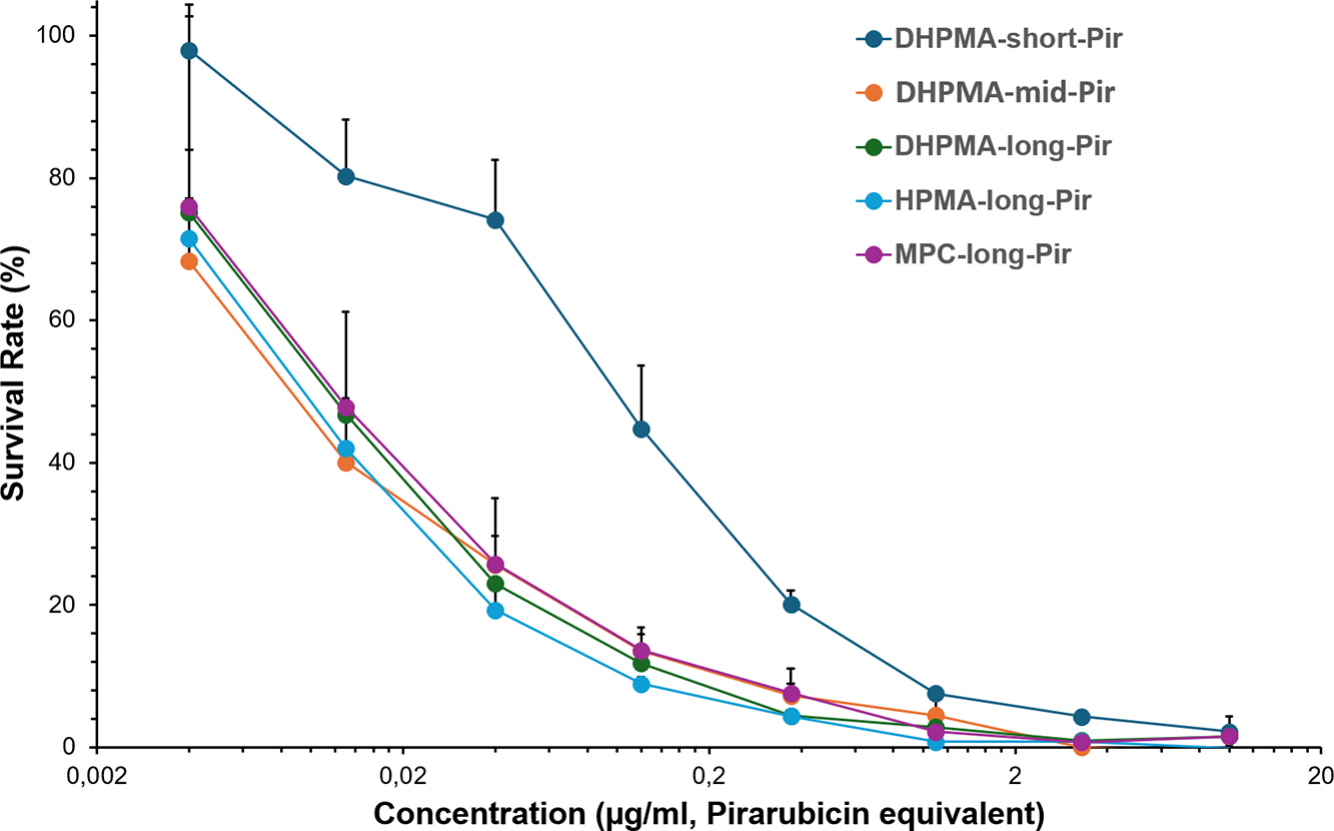
In vitro cytotoxicity of the polymer-Pir conjugates using mouse colon cancer C26 cells. Cells were plated in a 96-well plate at 5000 cells/well. After 24 h preincubation, indicated concentrations of different polymer-Pir conjugates were added, and the cells were treated for 48 h. Then, the MTT assay was performed. Data are mean ± SD.

### 3.6 Biodistribution of Pir in Mice

We hypothesized that the biodistribution of Pir delivered by various water-soluble polymers with distinct linker properties should be influenced by both the hydrophilicity of the polymer carrier and the pH-sensitivity of the linker. To evaluate this, we quantified the levels of released Pir in plasma, tumor (sarcoma S-180), kidney, and liver 24 h following intravenous administration (Figures 5 and S4).

**Figure 5:**
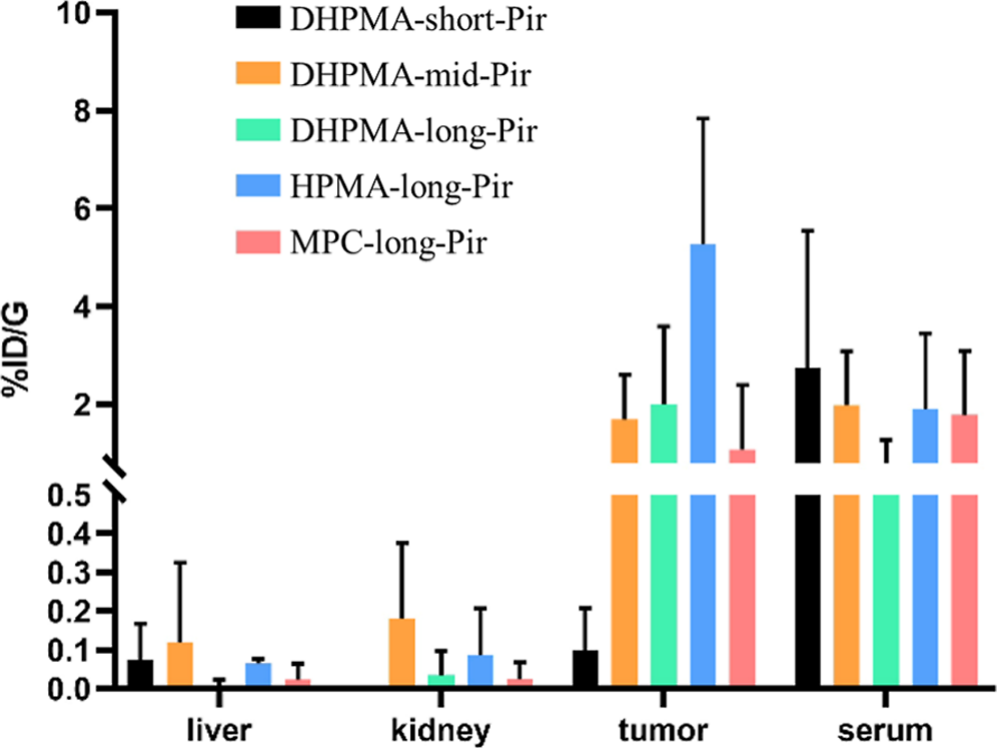
Biodistribution of polymer-Pir conjugates in mice 24 h following intravenous administration. **DHPMA-long-Pir** (green), **DHPMA-mid-Pir** (orange), **DHPMA-short-Pir** (black), HPMA-long-Pir (blue), and **MPC-long-Pir** (red). *n* = 4.

Importantly, all polymer conjugates exhibited very low uptake in the liver and kidneys, with levels below 0.2% of the injected dose per gram of tissue (%ID/g), highlighting their excellent biocompatibility. The accumulation of Pir delivered by the polymer conjugates in the liver, kidney, and serum was comparable (Figure S6), with observed differences within the standard deviation of the measurements. However, tumor accumulation of Pir was significantly lower for the **DHPMA-short-Pir** conjugate, with less than 0.2% ID/g, compared to approximately 2–6% ID/g for all other conjugates. We hypothesize that **DHPMA-short-Pir** may accumulate in other body tissues not examined in the present study. The absence of a linker between the polymer backbone and Pir likely may result in a more compact macromolecular structure, as indicated by its smallest hydrodynamic size (Table 3). This rigid architecture may reduce biocompatibility and increase the likelihood of accumulation in healthy organs. In contrast, the highest tumor accumulation was observed for the **HPMA-long-Pir** conjugate with the most flexible linker, demonstrating excellent potential for clinical tumor treatment.

### 3.7 Anti-Tumor Efficacy of the Polymer-Pir Conjugates

The therapeutic potential of the polymer-Pir conjugates was evaluated by using murine sarcoma S-180 as a tumor model in vivo. The conjugates were administered intravenously at a dose of 2.5 mg/kg Pir equivalent, representing approximately 3% of the maximum tolerated dose. This low dosage was chosen to highlight potential differences in antitumor efficacy among the conjugates.

The results (Figure 6) were consistent with those of the drug release and biodistribution studies. Conjugate **DHPMA-short-Pir**, with the lowest drug release rate and tumor accumulation, exhibited the least tumor growth retardation. In contrast, all of the other conjugates demonstrated excellent tumor growth inhibition, with no statistically significant differences among them. However, the conjugate **DHPMA-mid-Pir** (aminopropanoyl linker) showed slightly lower antitumor activity than conjugates with the long (aminohexanoyl) linker, which released Pir more rapidly. The highest antitumor activity was observed for conjugates with long linkers, **HPMA-long-Pir**, **DHPMA-long-Pir**, and **MPC-long-Pir**. However, the first two nanomedicines, **HPMA-long-Pir** and **DHPMA-long-Pir**, demonstrated significantly higher efficacy, resulting in a near-complete cure of sarcoma tumors at a dose of around 3% of the maximum tolerated dose. This finding highlights these nanomedicines as highly promising candidates for further preclinical evaluation.

**Figure 6:**
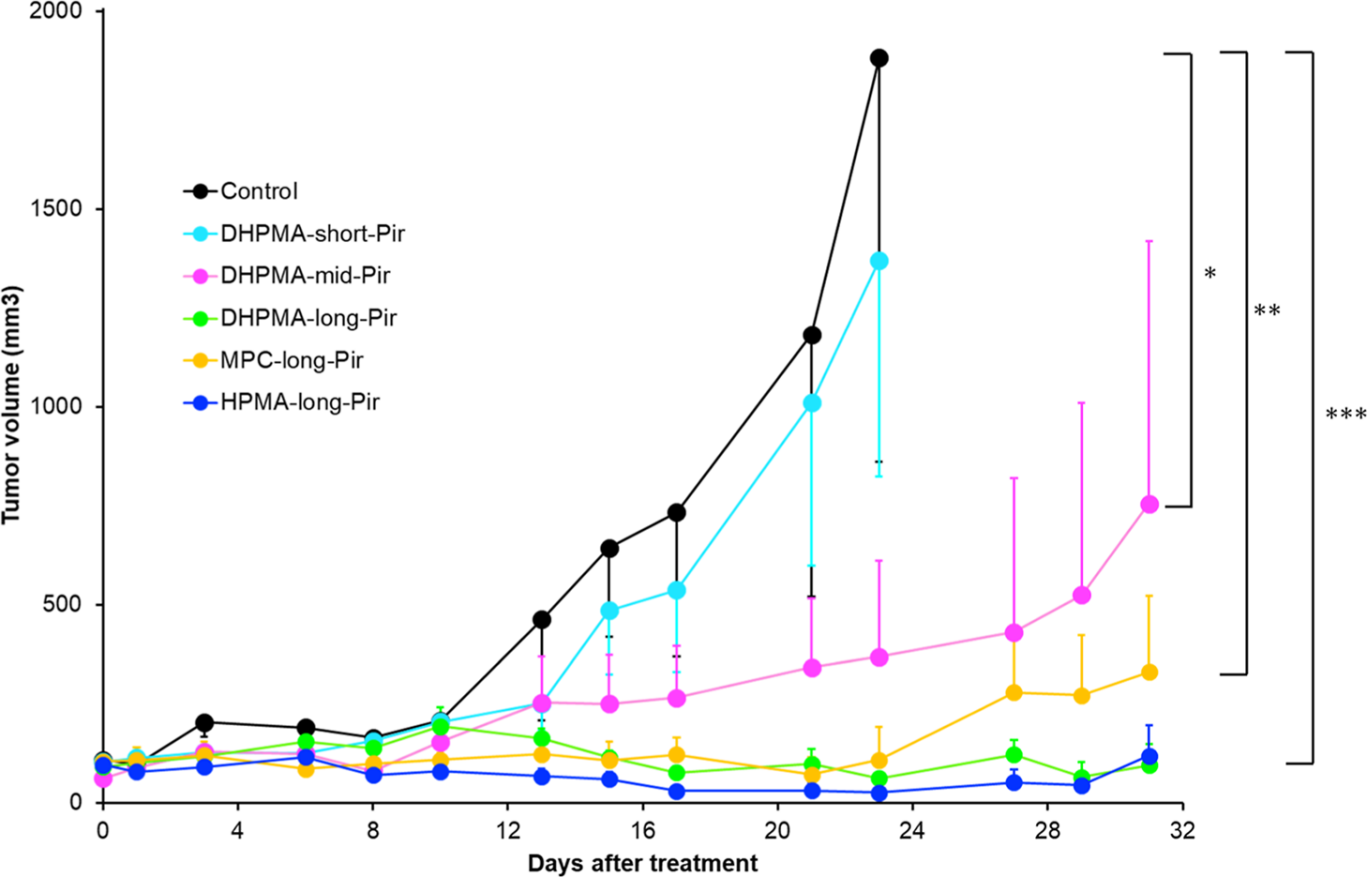
Anticancer efficacy of polymer-Pir conjugates with the pH-controlled release of Pir expressed as S-180 sarcoma growth retardation (dose 2.5 mg Pir eq kg^–1^ when the tumor grew to 6–8 mm in diameter). **DHPMA-long-PIR** (green), **DHPMA-mid-PIR** (red), **DHPMA-short-PIR** (blue), **HPMA-long-PIR** (blue), and **MPC-long-PIR** (orange). *n* = 8. *P < 0.05, **P < 0.01, ***P < 0.001.

In summary, within the studied set of polymer-Pir conjugates, the antitumor efficacy of the polymer therapeutics in vivo depended directly on the drug release rate, which is influenced by the linker structure between the hydrazone-bonded Pir and the polymer backbone. The most effective conjugates exhibited a high degree of pH sensitivity, characterized by minimal Pir release under neutral bloodstream conditions and rapid activation of Pir in a mild acidic tumor microenvironment. Moreover, the structural design of conjugates with a long linker demonstrated exceptional efficacy in solid tumor treatment, likely due to the superior hydrophilicity and biocompatibility of these polymer carriers.

## 4 Conclusions

Here, we deeply evaluated the influence of hydrophilic and biocompatible polymer-Pir conjugates design on drug release, biodistribution, and antitumor efficacy. The findings underscore the critical role of linker length between the polymer chain and hydrazone bond in controlling the hydrolytic release of Pir, with longer linkers facilitating faster drug release under acidic conditions, thereby enhancing the therapeutic efficacy. Conjugates with aminohexanoyl linkers demonstrated superior drug release and antitumor activity compared to those with shorter or absent linkers. Furthermore, the main chain polymer carrier structure slightly influenced the pH-sensitiveness, biodistribution, or therapeutic outcomes within the studied set, being optimal for the HPMA- and DHPMA-based polymer therapeutics. The MPC-based polymer system showed slightly elevated drug release at neutral conditions mimicking the bloodstream and the associated lower uptake in the tumor and lower therapeutic efficacy. Importantly, the DHPMA-based conjugates with the shortest linker between the polymer chain and hydrazone bond displayed markedly reduced tumor accumulation and therapeutic efficacy, thus excluding this system from further therapeutic testing.

Overall, the study confirms that the rate of drug release, dictated by the linker structure, is a pivotal determinant of therapeutic efficacy, whereas the polymer backbone’s hydrophilicity and size have a lesser impact. These findings provide a foundation for designing polymer–drug conjugates with optimized structures for enhanced anticancer performance.

## Supplementary Material



## Data Availability

The data that support the findings of this study are available from the corresponding author upon reasonable request.

## Notes

1H NMR spectra for polymer precursor and polymer conjugate; SEC chromatograms of polymer precursors and polymer conjugates; scheme of the synthesis of fluorescently labeled polymers; confocal microscopy images of PANC-1 cells after 6 h of incubation with fluorescently labeled homopolymers and its quantification; and biodistribution of polymer-Pir conjugates in mice (PDF)
