# Supplementary material for: Polymer Nanomedicines with pH-Triggered Pirarubicin Release: Revealing the Role of Carrier Hydrophilicity and Release Kinetics in Anticancer Performance
Source: Biomacromolecules. 2025 Sep 15;26(10):7013–23. doi: 10.1021/acs.biomac.5c01344 (PMC12522147; doi:10.1021/acs.biomac.5c01344)
Supplement: Supplementary file 1 [file bm5c01344_si_001.pdf]

Supplementary material

Polymer Nanomedicines with pH-Triggered Pirarubicin Release: Revealing the Role of Carrier Hydrophilicity and Release Kinetics in Anticancer Performance

Sára Pytlíková[a,#], Benchun Jiang[b,c,#], Lucie Woldřichová[a], Kevin Kotalík[a], Ladislav Androvič[a], Shanghui Gao[b], Vladimír Šubr[a], Anna Rumlerová[a], Robert Pola[a], Natália Podhorská[a], Marcela Filipová[a], Mingjie Zhang[b, d], Michal Pechar[a,], Jun Fang[b], Richard Laga[a], Tomáš Etrych[a,*]*

[a] *Institute of Macromolecular Chemistry, Czech Academy of Sciences, Heyrovského nám. 2, Prague 6, 162 00, Czech Republic*

[b] *Faculty of Pharmaceutical Sciences, Sojo University, Kumamoto 860 0082, Japan*

[c] *Department of Gastrointestinal Surgery, Shengjing Hospital of China Medical University, No.36 Sanhao Street, Shenyang 110004, Liaoning, China.*

[d] *Department of General Surgery, Shengjing Hospital of China Medical University, No.36 Sanhao Street, Shenyang 110004, Liaoning, China.*

[#] *contributed equally*

[*] *corresponding authors*

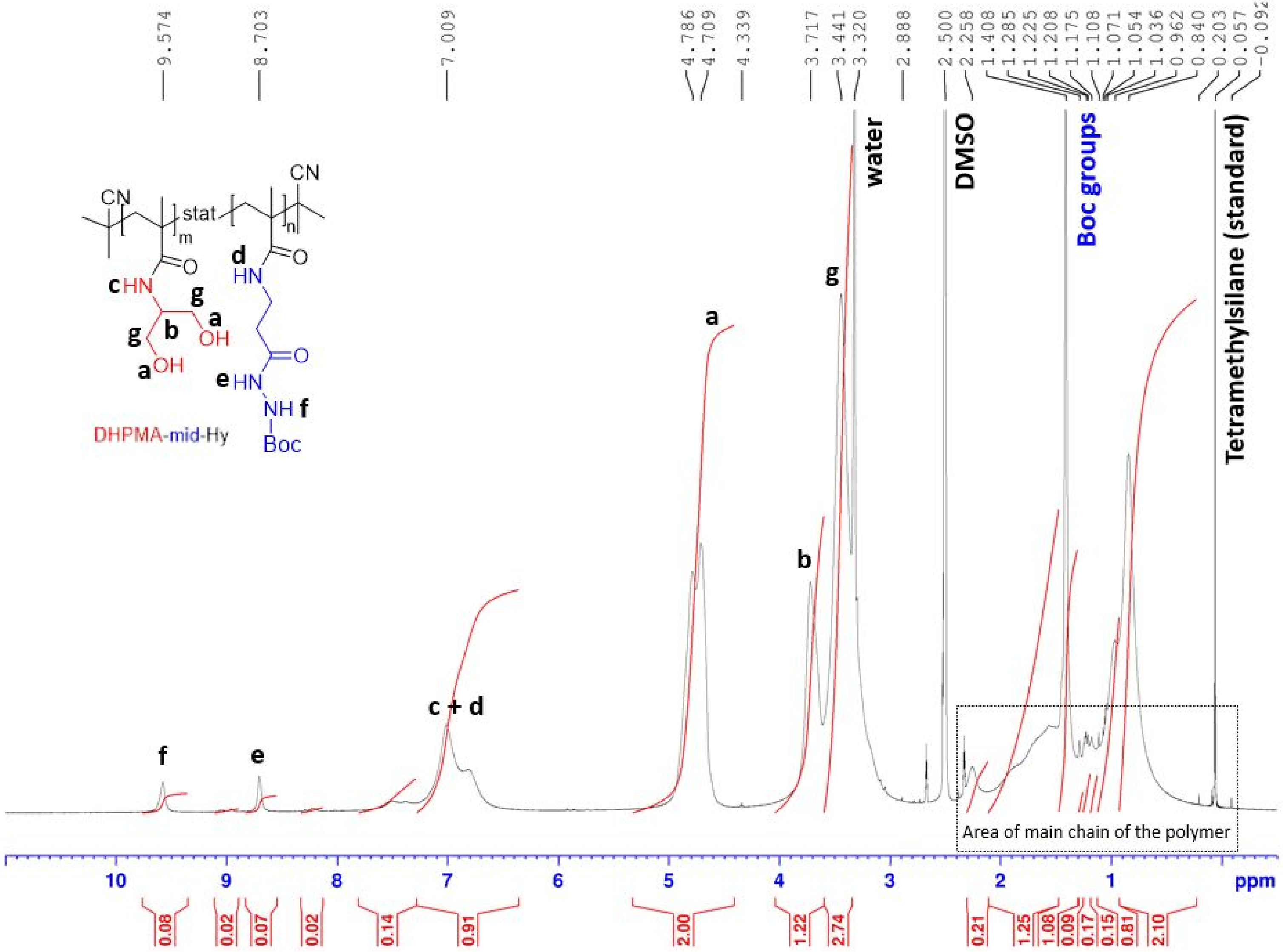

Figure S1: ^{1}H NMR spectra of the DHPMA-mid-Hy-Boc polymer precursor.

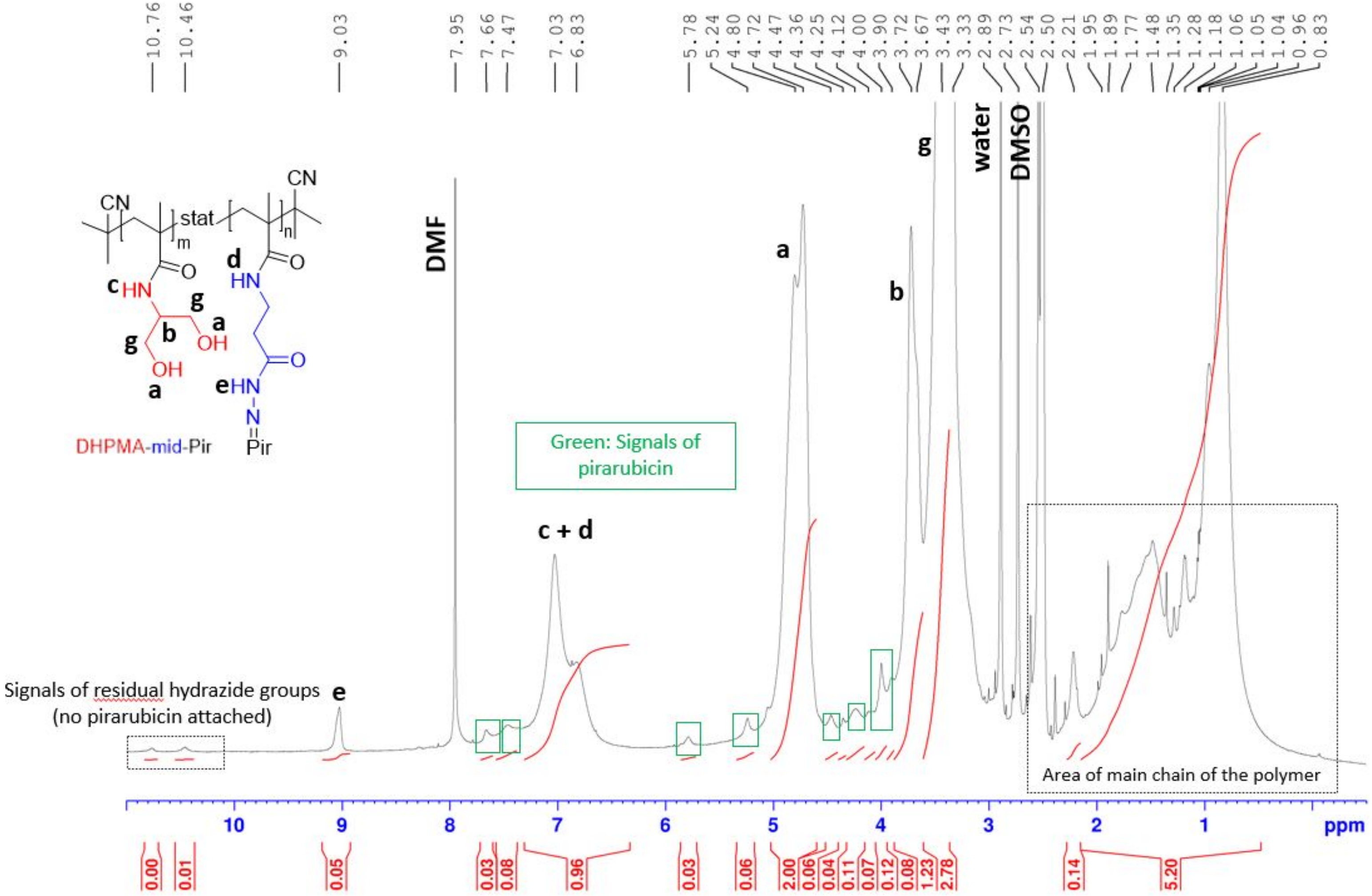

Figure S2: ^{1}H NMR spectra of the DHPMA-mid-Pir polymer conjugate. Spectrum of pirarubicin is depicted in: Pytlíková, Sára, et al. "Highly hydrophilic methacrylamide-based copolymers as precursors for polymeric nanomedicines containing anthracyclines." *European Polymer Journal* 205 (2024): 112756.

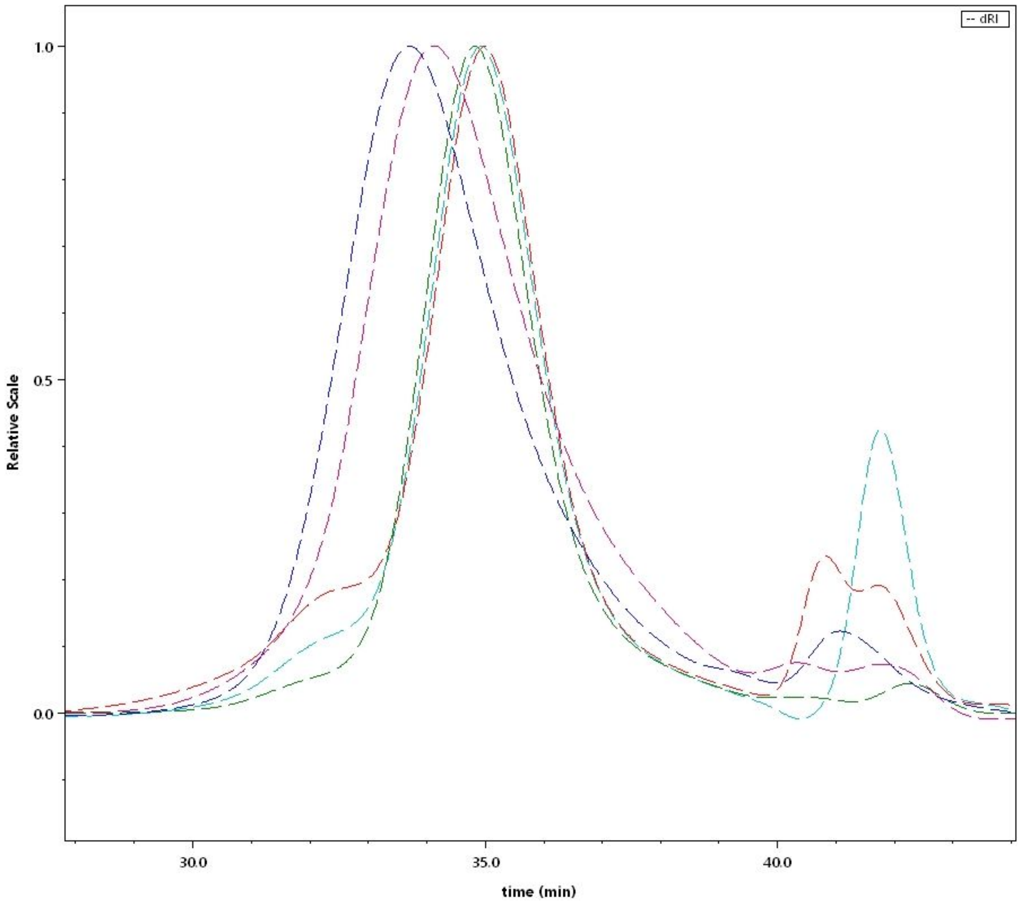

Figure S3A: SEC chromatograms with refractive index (RI) detection of copolymers with free hydrazide groups HPMA-long-Hy (Blue, dashed), MPC-long-Hy (Magenta, dashed), DHPMA-long-Hy (Red, dashed), DHPMA-mid-Hy (Green, dashed) and DHPMA-short-Hy (Cyan, dashed). Data measured on HPLC Shimadzu system equipped with Superose 6 Increase 10/300 GL column in PBS.

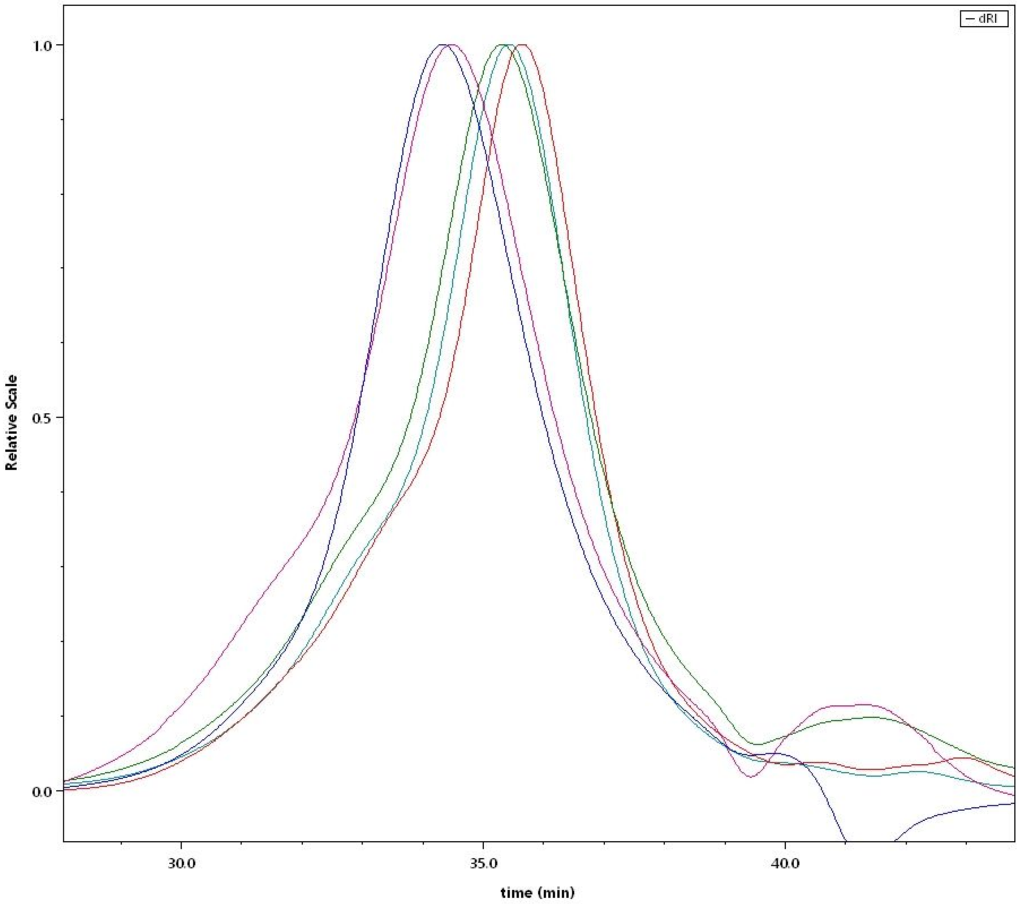

Figure S3B: SEC chromatograms with refractive index (RI) detection of polymer-Pir conjugates HPMA-long-Pir (Blue, solid), MPC-long-Pir (Magenta, solid), DHPMA-long-Pir (Red, solid), DHPMA-mid-Pir (Green, solid) and DHPMA-short-Pir (Cyan, solid). Data measured on HPLC system equipped with Superose 6 Increase 10/300 GL column in PBS.

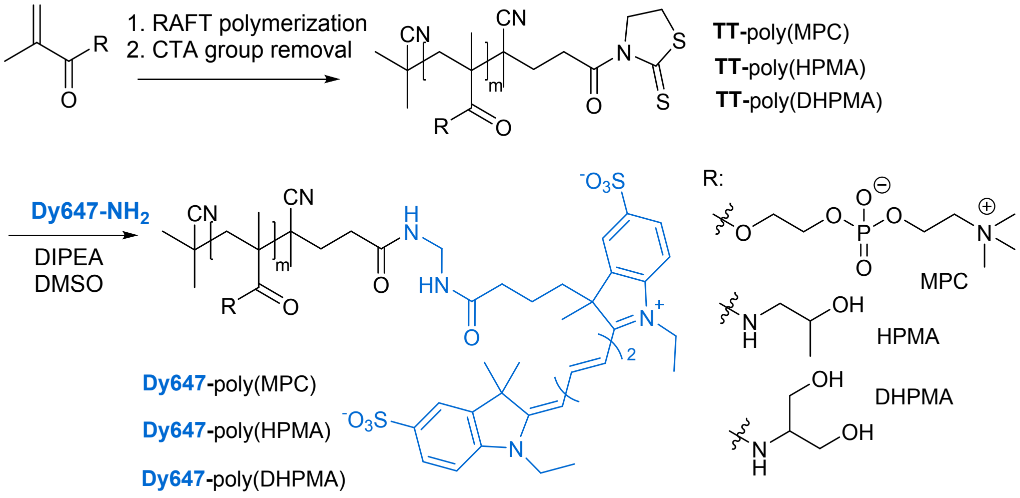

Scheme S1: Synthesis of semitelechelic homopolymers labeled by fluorescent dye Dy-647.

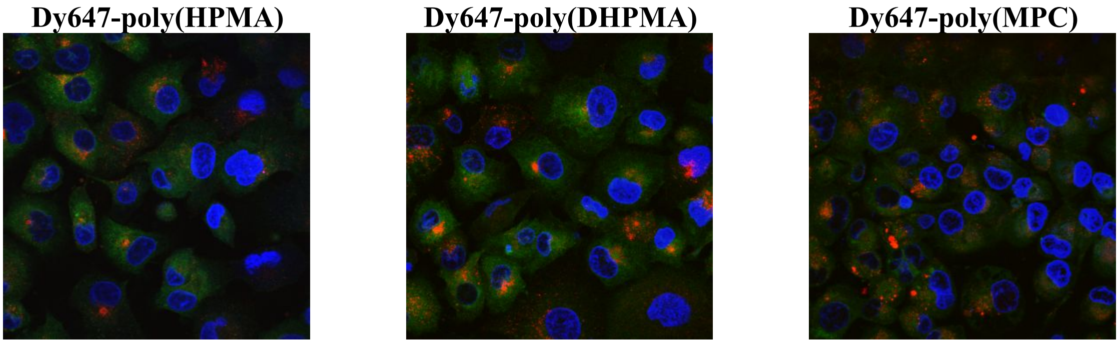

Figure S4: Confocal microscopy images of PANC-1 cells after 6 h of incubation with fluorescently labeled homopolymers (red) at concentration 1 µg/mL. Cell nuclei were stained with Hoechst 33342 nuclear stain (2 µg/ml, blue) and cell membranes with CellMaskTM (green).

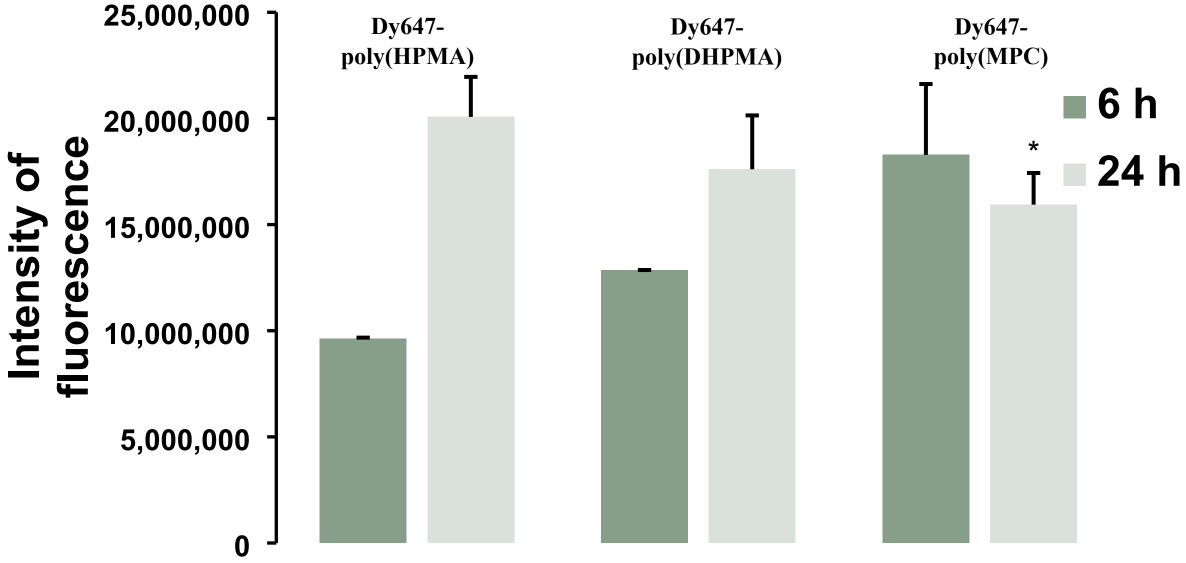

Figure S5: Quantification of fluorescence intensity of Dy647-labeled homopolymers internalized in PANC-1 cells after 6 h (dark green) and after 24 h (light green). * $p < 0.05$ was considered statistically significant compared to control poly(HPMA).

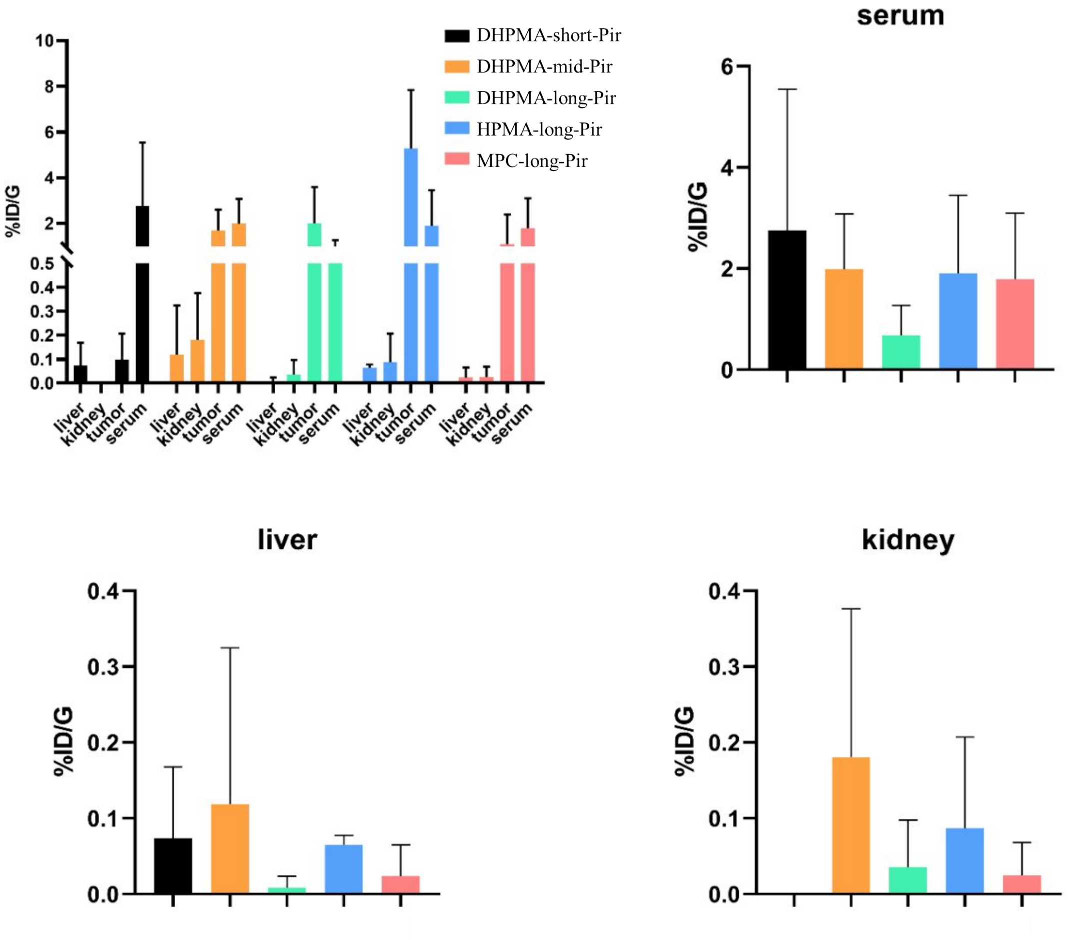

Figure S6: Biodistribution of polymer-Pir conjugates in mice.
